# CARING FOR FAMILY MEMBERS WITH ADVANCED DEMENTIA: MEN’S EXPERIENCES

**DOI:** 10.1093/geroni/igae098.0126

**Published:** 2024-12-31

**Authors:** Aimee Mooney, Charles Boardman, Sarah Gothard, Emily Tupper, Christina Zonker, Allison Lindauer

**Affiliations:** Oregon Health & Science University, Portland, Oregon, United States; Oregon Health & Science University, Portland, Oregon, United States; Oregon Alzheimer’s Disease Research Center, Oregon Health & Science University, Portland, Oregon, United States; Oregon Alzheimer’s Disease Research Center, Oregon Health & Science University, Portland, Oregon, United States; Oregon Alzheimer’s Disease Research Center, Oregon Health & Science University, Portland, Oregon, United States; Oregon Alzheimer’s Disease Research Center, Oregon Health & Science University, Portland, Oregon, United States

## Abstract

About one-third of dementia family caregivers are men, and this number is increasing due to societal changes, shifting family patterns, and financial drivers. Caregiving men are an understudied group, representing about 30% of participants in caregiving research. A better understanding of men in caregiving can facilitate personalization of interventions, increase research participation, and sustain retention. Here we describe a subgroup of men who participated in the intervention study, Tele-STELLA (Support via Technology: Living and Learning with Advancing dementia, R01AG067546). Tele-STELLA is a multi-component, psychoeducational intervention designed to facilitate effective management of behavioral symptoms in those with advanced dementia. Of the 150 Tele-STELLA caregivers in the sample, 37 (25%) were men. Burden was measured using the Revised Memory and Behavior Problems Checklist reactivity subscale. Using a two-tailed t test at 0.1 α, we found that there was significant improvement in reactivity scores (burden) for the men that completed the intervention (n=27). Depression did not change over the 8-week intervention period despite disease progression in their family members with dementia. Ten Tele-STELLA men participated in focus groups post-intervention. Employing thematic analysis, three important themes emerged. First, the men signed up for the study because it was recommended by their health care professionals. Second, men found the solution-focused curriculum increased their sense of “capability” as caregivers, and third, Tele-STELLA provided a sense of camaraderie with their caregiving men peers. Future studies may focus recruitment strategies on informing health care providers, tailor interventions to be solution-focused, and foster communication between men to build solidarity.

